# Seasonal occurrence and individual variability of bull sharks, *Carcharhinus leucas,* in a marine reserve of the southwestern Gulf of California

**DOI:** 10.7717/peerj.17192

**Published:** 2024-05-16

**Authors:** Frida Lara-Lizardi, Eleazar Castro, Vianey Leos Barajas, Juan Manuel Morales, Edgar Mauricio Hoyos-Padilla, James Ketchum

**Affiliations:** 1 Pelagios Kakunjá, La Paz, Baja California Sur, Mexico; 2 Orgcas, La Paz, Baja California Sur, Mexico; 3 Migramar, Bodega Bay, CA, United States of America; 4 Centro Interdisciplinario en Ciencias Aplicadas de Baja California Sur A.C., La Paz, Baja California Sur, Mexico; 5 Department of Statistical Sciences, University of Toronto, Toronto, Canada; 6 School of the Environment, University of Toronto, Toronto, Canada; 7 Department of Statistics, North Carolina State University, North Carolina, United States of America; 8 Department of Forestry and Environmental Resources, North Carolina State University, North Carolina, United States of America; 9 Grupo de Ecología Cuantitativa. INIBIOMA, Universidad Nacional del Comahue, Bariloche, Argentina; 10 School of Biodiversity, One Health & Veterinary Medicine, University of Glasgow, Glasgow, Scotland; 11 Fins attached: Marine Research and Conservation, Colorado Springs, CO, United States of America; 12 Centro de Investigaciones Biológicas del Noroeste (CIBNOR), La Paz, Baja California Sur, Mexico

**Keywords:** Acoustic telemetry, Temporal patterns, Sexual segregation, Probabilistic models, Movement ecology, Bull sharks, Cabo Pulmo, Bayesian models, Residency patterns, Residency probability

## Abstract

**Background:**

Studying how the bull sharks aggregate and how they can be driven by life history traits such as reproduction, prey availability, predator avoidance and social interaction in a National Park such as Cabo Pulmo, is key to understand and protect the species.

**Methods:**

The occurrence variability of 32 bull sharks tracked with passive acoustic telemetry were investigated via a hierarchical logistic regression model, with inference conducted in a Bayesian framework, comparing sex, and their response to temperature and chlorophyll.

**Results:**

Based on the fitted model, occurrence probability varied by sex and length. Juvenile females had the highest values, whereas adult males the lowest. A strong seasonality or day of the year was recorded, where sharks were generally absent during September–November. However, some sharks did not show the common pattern, being detected just for a short period. This is one of the first studies where the Bayesian framework is used to study passive acoustic telemetry proving the potential to be used in further studies.

## Introduction

Acoustic telemery offers the capacity for long-term monitoring, facilitating the tracking of individual sharks over extended durations (Jacoby, Croft & Sims, 2012). Utilizing coded transmitters and an array of acoustic receivers enables the characterization of aggregations formed through habitat selection, influenced by abiotic factors (Jacoby & Piper, 2023). These environmental fluctuations may serve as cues for movements and migrations (Schlaff, Heupel & Simpfendorfer, 2014). This method yields valuable insights into seasonal migrations, site fidelity, and the utilization of specific habitats within marine reserves (Hearn et al., 2010; Ketchum et al., 2014a). Integrating this technique with innovative analytical approaches, such as Bayesian models, allows for the integration of diverse sources of information and the estimation of unknown parameters across different levels of the model. The model is designed to accommodate various sources of uncertainty (Carpenter et al., 2017), including imperfect detection of tagged sharks, individual variability in movement behavior, and environmental factors influencing shark movement patterns (Heupel & Simpfendorfer, 2007).

Through the incorporation of additional data sources, such as environmental factors (*e.g.*, temperature, chlorophyll) and biological variables (*e.g.*, maturity stage, gender, prey abundance), along with prior knowledge about population structure, the Bayesian model can effectively capture the intricate interactions between environmental conditions and shark movement behavior. This variability implies that individuals within a group may exhibit different behaviors even when exposed to identical environmental conditions (Daly et al., 2014).

Understanding the seasonal occurrence of bull sharks (*Carcharhinus leucas*) is crucial for assessing the temporal dynamics of their presence within a protected area. The documentation of their occurrence throughout the year provides important insights into their ecological needs and the potential drivers influencing their movements (Simpfendorfer & Heupel, 2012; Daly et al., 2014). Bull sharks are distinguished by their exceptional adaptability and extensive habitat range, spanning coastal waters, estuaries, and even upstream river systems (Daly et al., 2014). They are distributed in tropical and subtropical waters, in coastal waters, sometimes in brackish and freshwater rivers (Compagno, 2001). They reach up to 400 cm in total length (TL), with males maturing at 157–226 cm and females at 180–230 cm, producing pups ranging from 60–80 cm in length (Branstetter, Musick & Colvocoresses, 1987). In the latest assessment by the International Union for Conservation of Nature (IUCN), the species is categorized as “Near Threatened” globally (Rigby et al., 2021).

The southwestern Gulf of California, known for its diverse marine ecosystems and the establishment of no take marine reserves, such as Cabo Pulmo National Park (CPNP; Aburto-Oropeza et al., 2011), offers an optimal setting for investigating the seasonal movements and distinct behaviors of bull sharks. The interplay of warm waters from the Costa Rica Current in summer and cold waters from the California Current in winter as they enter the Gulf of California shapes its dynamic oceanography (Valle-Rodríguez & Trasviña Castro, 2017). Consequently, the region hosts a variety of sub-tropical and tropical species at different times of the year (Sundström et al., 2001). Detailed studies focusing on bull shark occurrence in areas like Cabo Pulmo and individual variability in specific regions are imperative for formulating effective conservation and management strategies (Espinoza et al., 2014). Hence, the objectives of this study were: (1) to assess the behavioral variability among individual bull sharks considering the differences by sex and length, and (2) to examine how environmental variables, water temperature and chlorophyll, influence the occurrence patterns of bull sharks within and beyond the CPNP.

## Materials and Methods

**Figure 1:**
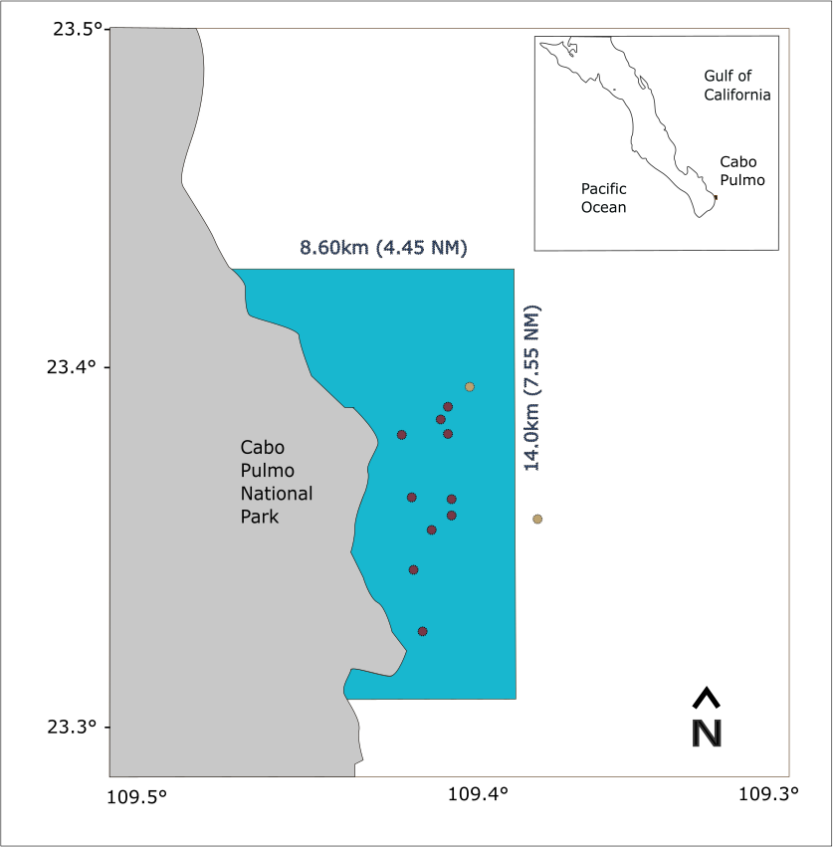
Study area map showing the location of CPNP in the southwestern area of the Gulf of California. The blue polygon indicates the extension of the national park, red dots indicate the shallow receivers and yellow dots, the deep receivers.

CPNP is located off the southern tip of the Baja California peninsula (23°25′N, 109°25′W; see Fig. 1), at the entrance of the Gulf of California, where tropical and subtropical waters mix (Zaitsev et al., 2014). The area provides the conditions for a highly biodiverse system with species of different biogeographic provinces and the northernmost coral reef in the eastern tropical Pacific (ETP; Cortés et al., 2017).

CPNP was designated as a no-take national marine park in 1995 (Reyes-Bonilla et al., 2014). Within 10 years of the park’s creation, fish biomass was reported to have increased by 463% and biomass of top predators increased 11-fold (Aburto-Oropeza et al., 2011). The park covers an area of 71 km^2^ and had 25 km^2^ of no-take area within it, however it the core areas or no-take have increased to near 100% in recent years. It was declared a UNESCO World Heritage site in 2005 and a Ramsar Site in 2008. Reyes-Bonilla et al. (2014) determined that 5,300 tons of the park’s animal biomass is exported to adjacent areas per year.

This study was carried out under permits from the general auspices of DGVS (Dirección General de Vida Silvestre), SEMARNAT (Secretaría del Medio Ambiente y Recursos Naturales), the Secretaría de Agricultura, Ganadería, Desarrollo Rural, Pesca y Alimentación (DGOPA.06668.150612.1691) and Comisión Nacional de Áreas Naturales Protegidas, CONANP (F00.DRPBCPN-APFFCSL.REBIARRE-102/13). These are the relevant Mexican authorities governing all research actions on wildlife and protected animals and areas in Mexico. The protocol used for this study therefore complied with all the relevant national, international, and institutional guidelines regarding animal care, following the Institutional Animal Care and Use Active Protocol #16022 from the University of California, Davis.

The study period was from 2015 to 2019. Individually coded transmitters were placed on bull sharks and they were detected by 16 autonomous receivers deployed in CPNP from 2015 to 2019. The transmitters (V16; Vemco Ltd., Halifax, Nova Scotia, Canada) were placed on 44 bull sharks, however only 32 were used for the analyses (Table S1). The transmitters had a transmission frequency of 69 kHz, a power of 152–158 dB re 1 µPa 1 m, nominal delay of 60 to 180 s, and a battery life of 5–10 years. Transmitters were attached externally to sharks while scuba and freediving with spearguns by inserting a stainless-steel barb into the dorsal musculature at the base of the dorsal fin. The gender was determined by footage taken from cameras (GoPro Ltd., San Mateo, CA, USA) mounted on the speargun noting presence or absence of claspers, and their stage of maturity (juvenile, sub-adult, adult), as estimated by comparing their size to that of the taggers. The life stage was included to assess if all life stages were represented in the observations. This study did not consider the growth, limiting the study period to 3 years, assuming that sharks would not have significant differences in their behaviours.

An array of 16 acoustic receivers (VR2W; Vemco Ltd.) was maintained throughout CPNP to monitor the movements of these sharks. The mooring system used for the acoustic receivers was a buoy, a 2 m long rope and either a chain attached to a rock or crevice on the bottom or a sand anchor buried on the sand. Records of detections were examined and filtered with VUE 2.2.2 (VEMCO Software) for false positive and subsequently analysed with R (Version 3.2.4; R Core Team, 2016). The range of detection of the receivers was established to be 250–300 m. The technique used in such tests was to move away from a receiver moored at the bottom by increasing distances of 25 m (see Hearn et al., 2010). The percentage of detections were then expressed as a percentage of the number detected when the transmitters were directly above the receiver. The range tests were conducted during seas less than 1 m in height (Beafort scale = 3).

Individual tagged sharks were recorded from first tagged to last detected, *i.e.,* time at liberty (Hearn et al., 2010). Individuals were considered to be present in the study area if more than one detection was recorded on any receiver in the area on a given day. The number of days that each individual was present in CPNP over the study period was plotted on a timeline and categorized as ‘days detected’. A database was generated by integrating the acoustic detections into a daily summary where each row represents the behaviour of an individual on a given day. The degree of occurrence (DO) is defined as the number of days detected divided by duration of detections for that tag. A function was integrated to determine if the individual was present or not on a given day: present (1) if it had at least two detections in an interval of less than 15 min (see Ketchum et al., 2014b), or absent (0) if less than two detections in an interval of less than 15 min. A second function was integrated to determine their occurrence time in seconds (s), where only the seconds of each record with less than 15 min between them were added. The environmental variables were obtained from satellite imagery (MODIS and SeaWiFS).

Presence is determined by having at least two detections within a span of 15 min on any given day. Let *Y*_*t*_ be the random variable connected to the presence or absence of a shark on day *t*. As our primary objective is to relate a set of *J* environmental covariates, ***x*** = (*x*_1_, *x*_2_, …, *x*_*J*_), to the presence or absence of a bull shark in the Cabo Pulmo National Park, we formulated an initial model for the presence of an individual shark by allowing *Y*_*t*_ to be distributed according to a Bernoulli distribution with time(day)-specific probability *p*_*t*_. Bernoulli distribution can be used to describe events that can only have two outcomes, that is, success or failure (Carpenter et al., 2017). We further allowed *p*_*t*_ to be a function of ***x***.

\begin{eqnarray*}{Y}_{t}& \sim Bernoulli \left( {p}_{t} \right) \end{eqnarray*}

\begin{eqnarray*}\text{logit} \left( {p}_{t} \right) & ={\beta }_{0}+\sum _{j=1}^{J}{\beta }_{j}{x}_{j,t} \end{eqnarray*}

As CPNP provides an critical for the life history of species, we were primarily interested in relating the environmental seasonality to presence/absence of the bull sharks to understand the relationship between these factors and occurrence patterns. As such, our covariates are two trigonometric functions to relate the effect of day of the year, as well as chlorophyll and temperature (in C): ${x}_{1}=\cos \left( \frac{2\pi t}{365} \right) $, ${x}_{2}=\sin \left( \frac{2\pi t}{365} \right) $, ${x}_{3}=\log \left( chlorophyll \right) $ and ${x}_{4}=temperature \left( inC \right) $. We further centre each covariate value to *approximately* zero by specification of baseline values, ***x***^***B***^, for all ***x***, if needed. The model therefore takes in ${\mathbi{x}}_{t}^{\ast }= \left( {x}_{1}-{x}_{1}^{B},{x}_{2}-{x}_{2}^{B},\ldots ,{x}_{J}-{x}_{J}^{B} \right) $, where ${\mathbi{x}}^{\mathbi{B}}= \left( 0,0,0,28 \right) .$The parameters *β*_1:*J*_ are now interpreted as effects of deviations from the baseline values ${x}_{j}^{B}$, for *j* = 1, …, *J*.The slope *β*_0_ represents the baseline effect on occurrence probability.

The sharks varied in length and sex, which could play an important role in the occurrence patterns exhibited. As these are not environmental factors, we accounted for heterogeneity across individuals by extending the model so that the intercept term was a function of length and sex. Sex was included as an indicator variable corresponding to a value of 1 if the shark was male, and 0 if female. Length was further centred at approximately zero by subtracting 220 cm from the length of all sharks, which also represents an approximate upper bound at which sharks of both sexes reach maturity. Given sex and length, we expressed the intercept term for the *I* = 32 sharks as a linear combination of fixed effects *α*, sex and length, ${\beta }_{0,i}={\alpha }_{0}+{\alpha }_{1}mal{e}_{i}+{\alpha }_{2} \left( lengt{h}_{i}-220 \right) .$Our joint model thus far for all sharks is only allowed to vary across individuals by the intercept term. However, to account for differences across the effects of the environmental covariates as they relate to each shark, we further introduced hierarchical structures into the slope terms,

\begin{eqnarray*}{\beta }_{j,i}\sim N({\mu }_{j},{\sigma }_{j}) \end{eqnarray*}

where *μ*_*j*_ denotes the population level mean effect of ${x}_{j}^{\ast }$. Our joint model can be fully expressed as,

\begin{eqnarray*}{Y}_{i,t}& \sim Bernoulli \left( {p}_{i,t} \right) \end{eqnarray*}

\begin{eqnarray*}logit \left( {p}_{i,t} \right) & ={\beta }_{0,i}+\sum _{j=1}^{J}{\beta }_{j,i}{x}_{j,i,t}^{\ast } \end{eqnarray*}

\begin{eqnarray*}{\beta }_{j,i}& \sim N({\mu }_{j},{\sigma }_{j}) \end{eqnarray*}

\begin{eqnarray*}{\beta }_{0,i}& ={\alpha }_{0}+{\alpha }_{1}mal{e}_{i}+{\alpha }_{2} \left( lengt{h}_{i}-220 \right) \end{eqnarray*}

To take into account that the sex of one shark was not recorded, we modified the model formulation for that individual by introducing a parameter *π* ∈ [0, 1] that accounted for the probability that the shark was male, and thereby allowed for marginalization over the effect of sex, ${Y}_{i,t}\sim \pi Bernoulli \left( {p}_{i,t}{|}male \right) + \left( 1-\pi \right) Bernoulli \left( {p}_{i,t}{|}female \right) $.

In order to conduct inference in a Bayesian framework, we assigned informative prior distributions to all parameters of interest: *α*_0_, *α*_1_, *α*_2_, *μ*_1_, *μ*_2_, …, *μ*_*J*_, *σ*_1_, *σ*_2_, …, *σ*_*J*_, *π*. We used the software Stan designed by Carpenter et al. (2017) to draw samples from the joint posterior distribution of both the population-level and individual-specific effects, $p \left( \alpha ,\mu ,\sigma ,{\beta }_{\mathbi{i}}{|}\mathbi{y} \right) $. We used four chains and drew 2,000 samples from the joint posterior distribution, 1,000 of which were used as warmup, and assessed convergence by checking that all $\hat {R}\approx $ 1. For model checking, we examined using the method proposed by Gelman et al. (2013): the binned residuals, as well as inspected individual level fitted curves.

## Results

Forty-four bull sharks were tagged in CPNP from 2015–2019, one shark was tagged in 2015 (sex unknown), 20 in 2016 (15 females, five males), five in 2017 (one female, four males) and 18 in 2018 (10 females, eight males). Sharks varied between 160 cm TL and 280 cm TL.

Only 32 sharks were detected within the acoustic array. 21 sharks were detected in 2015–2016 and four of these returned to the park in 2017–2019, leading to four monitoring periods that spanned over 1 year in length. The mean DO was 0.365 (SD = 0.2143), which means that sharks were present 36.5% of the monitoring period. The maximum occurrence recorded was for a female juvenile shark of 160 cm TL that was present 73.7% of the time. The minimum DO was for a sub-adult male of 180 cm TL, who was present only 13.7% of the monitoring period.

**Figure 2:**
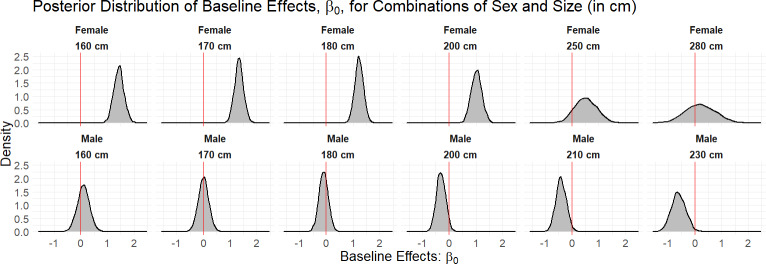
Density curves of the marginal posterior distributions for unique combinations of sex and size (in cm) observed in the data set. The red line at zero indicates the value of the baseline effect that leads to a residence probability of 50%, were no other terms in the model included. Values to the right of the red line lead to a residence probability greater than 50%, and vice versa for values to the left of the red line.

We fitted the hierarchical logistic regression model to 32 bull sharks with monitoring periods of at least 30 days in CPNP. To obtain draws from the joint posterior distribution of our parameters, we used three chains of 4,000 draws each, half of which were used for the warmup phase and checked multiple MCMC diagnostics that could point to issues with the sampling performance. Across all parameters, we had $\hat {R}=1$ sufficiently large bulk and tail effective sample size given the number of draws, and, overall, there were no Hamiltonian Monte Carlo-specific warnings, *e.g.*, divergences, Bayesian fraction of missing information. According to the fitted model, sex and length presented dissimilar occurrence probability across the years. Figure 2 shows that female sharks, in general, tend to have a higher baseline effect of occurrence, *β*_0,*i*_, than the male sharks, in particular at lengths less than or equal to 200 cm TL.

**Figure 3:**
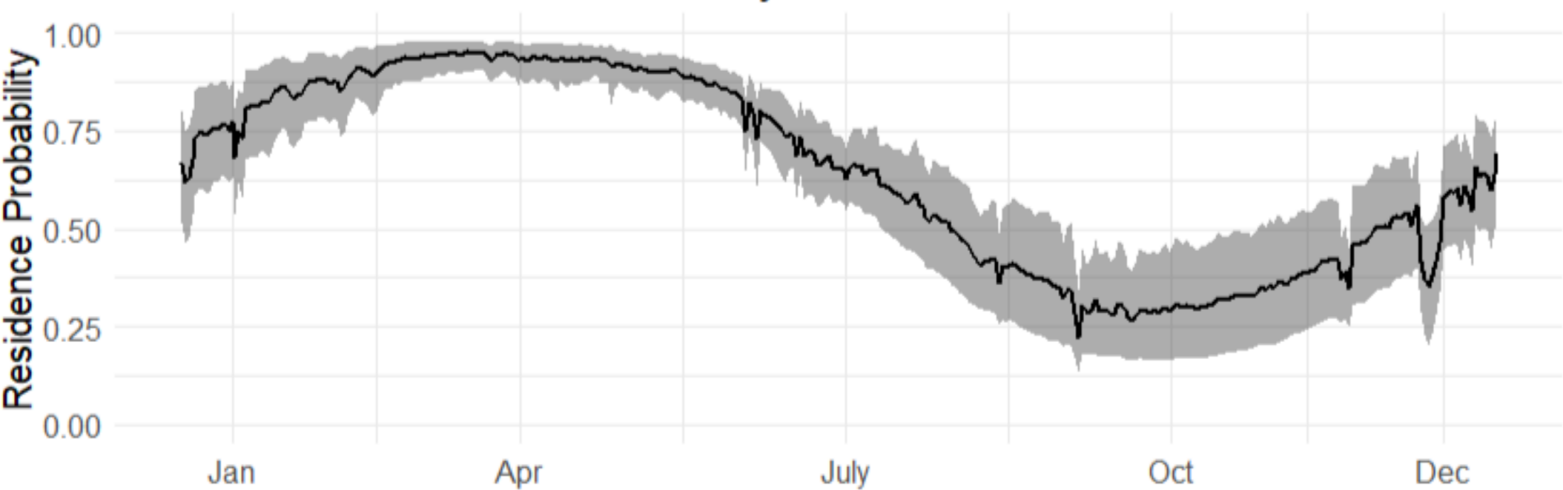
Marginal occurrence probability of bull sharks, averaged across all sexes, sizes and environmental covariates, in CPNP across the years (2016–2019). The 95% credible intervals are given in grey.

The marginal occurrence probability of bull sharks in Cabo Pulmo across an average year (2015–2019) showed the highest occurrence probabilities from March to May (Fig. 3). The months of September to November had the lowest occurrence probabilities but exhibited much more variability (grey shaded line) as not all sharks left during this period, although most of the sharks did leave.

**Figure 4:**
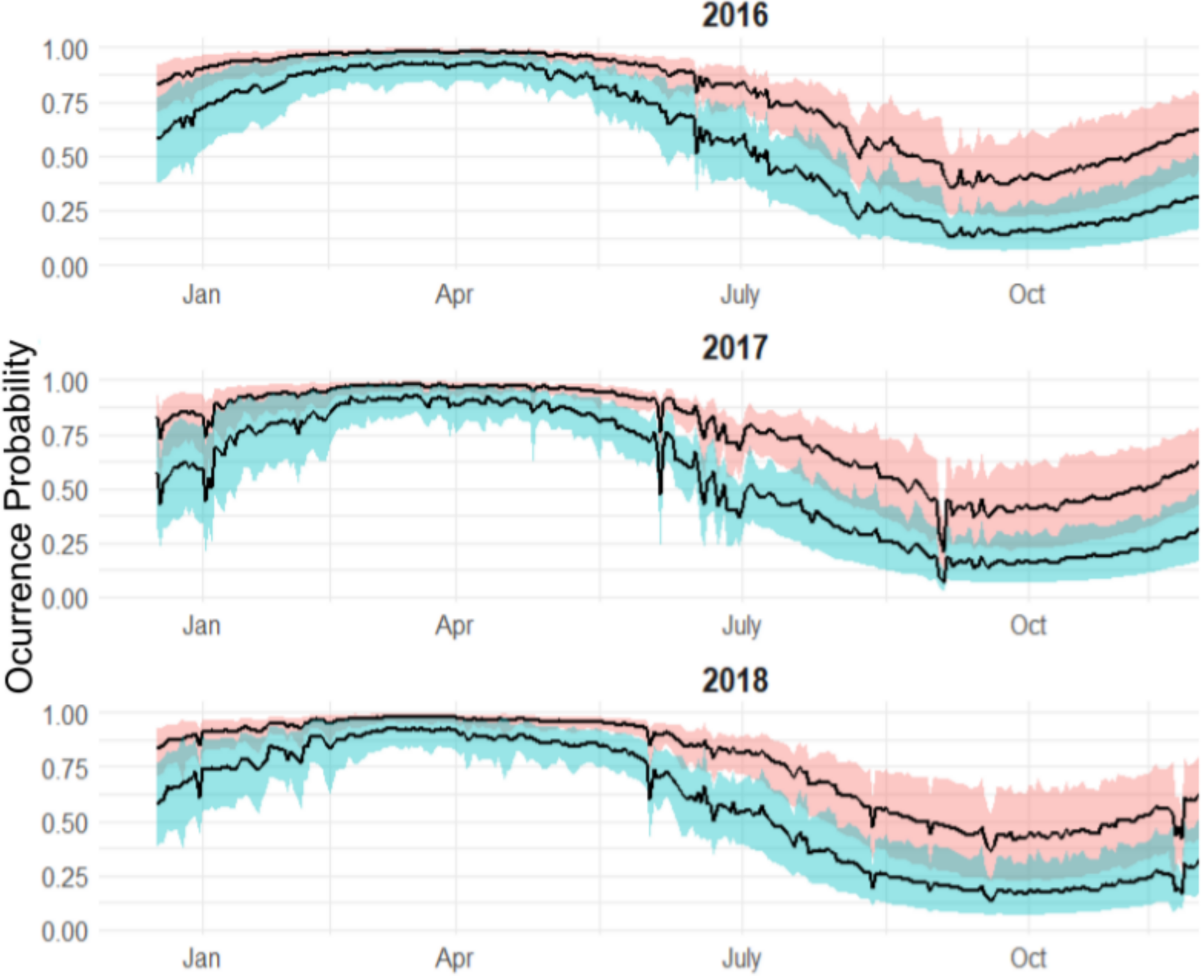
Expected occurrence probability for bull shark across sexes in 2017 given a length of 180 cm TL and the environmental covariate values observed in that year along with 95% credible intervals. Red shaded line represents the variation of the expected occurrence probability for the females and blue shaded for the males.

**Figure 5:**
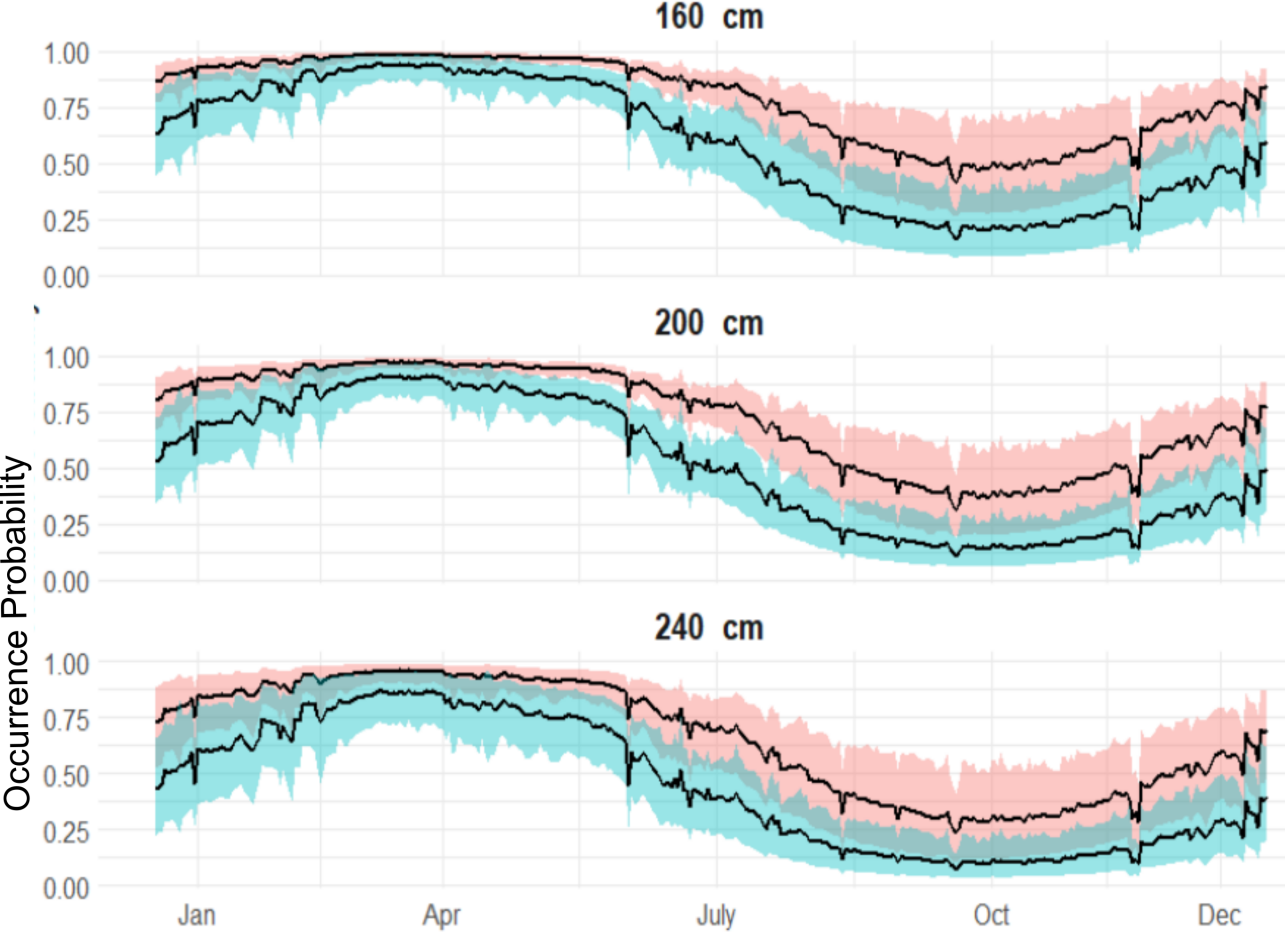
Expected sex-specific differences in occurrence probabilities across sizes in 2018. Red shaded line represents the variation of the expected occurrence probability for the females and blue shaded for the males.

Both sexes were likely to be present in April, whereas by October both sexes were not as likely to be present (Fig. 4), although the occurrence probability for females is higher than males year-round. We can further compare probabilities across sizes and sex in October, and at other times of the year (Fig. 5). Females were 30% more likely to be present than males for shark sizes of 240 cm TL. In general, differences across sex are much higher from July to the beginning of January, whereas in the spring, both sexes behave similarly.

**Figure 6:**
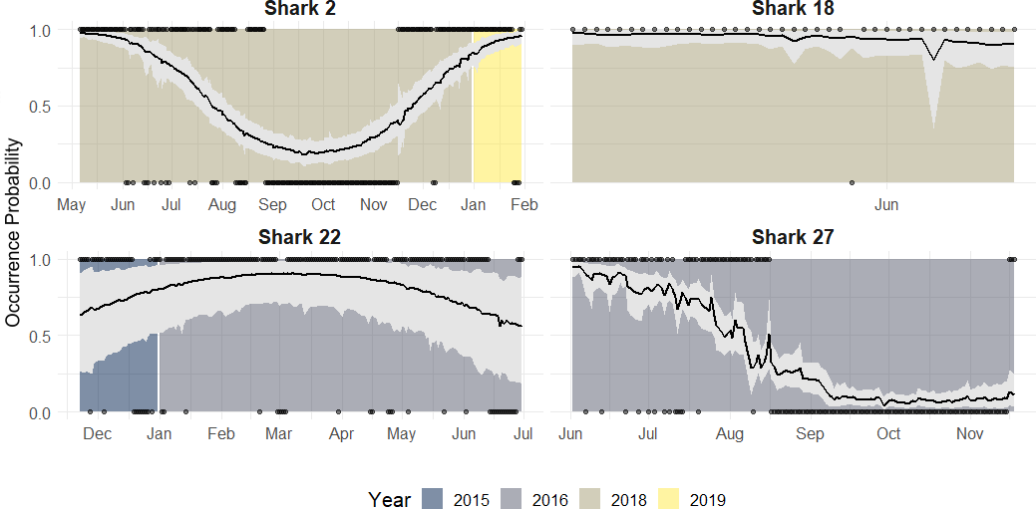
Occurrence probability curves for the bull shark and 95% credible intervals, along with the presence/absence data, denoted as 1 or 0, across the monitoring period (2015–2019).

Along with population level estimates, the hierarchical component of our model allowed for estimation of individual-level occurrence probability curves to each shark’s data. According to the individual occurrence patterns, 32 sharks showed four general types of behaviours during a year across the study period (Fig. 6):

1. Reduced occurrence during September-November (*i.e.,* Shark 2): Most of the individuals that were monitored for at least 12 months, showed this pattern.
2. High occurrence during the first months of the year followed by a general period of absence: (*i.e.,* Shark 27): Some sharks showed a high occurrence during the first period of the year after tagging (May) and by September they are nearly all absent and do not tend to be detected during the monitoring period.
3. Sharks are present/highly resident the whole year (*i.e.,* Shark 22): There is no evidence that these individuals leave the park across the years.
4. No clear pattern due to the short monitoring period (*i.e.,* Shark 18): Some sharks are detected for a short time, so there is no evidence of seasonality. They could have either shed the tag or left the park.

**Figure 7:**
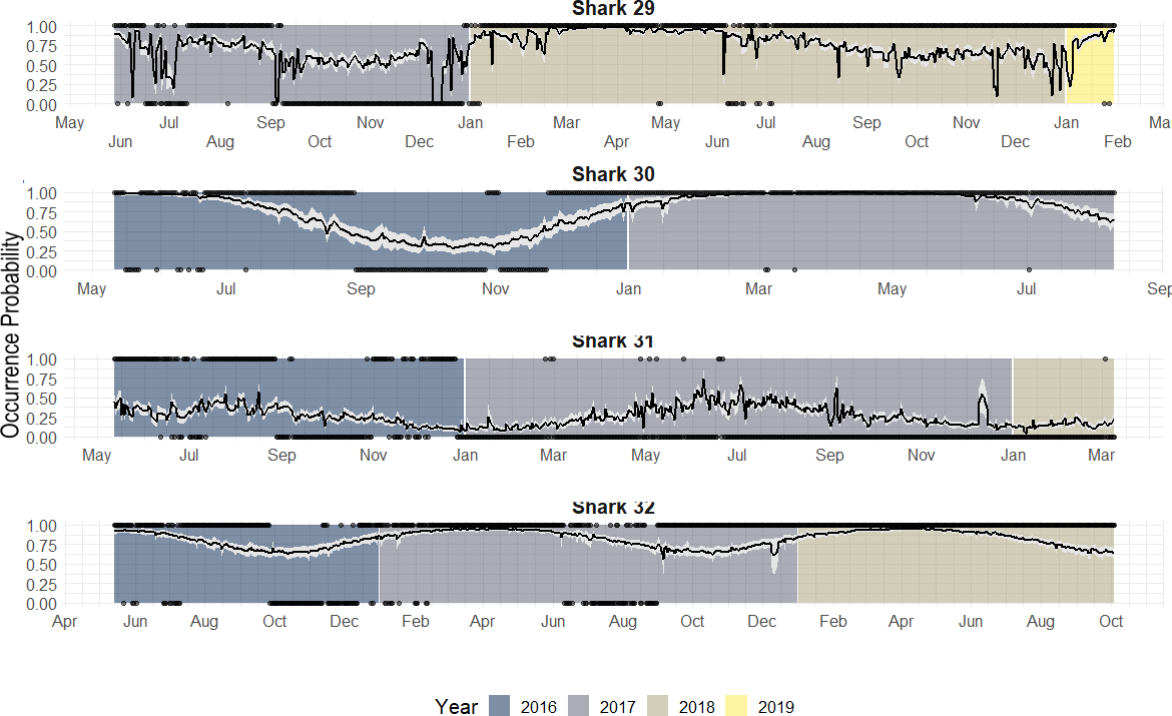
Predicted occurrence probability curves for the four bull shark with presence/absence data across the monitoring period (2016–2018) along with the presence/absence data, denoted as 1 or 0, across the monitoring period.

Individuals that were monitored over multiple years showed different patterns (Fig. 7):

1. Highly resident across the year (*i.e.,* Shark 29): This shark was tracked for a period longer than 2 years. It showed a high occurrence probability the first months of the year, and during the summer months, the probability was close to 50% in both years.
2. Reduced occurrence during September-November (*i.e.,* Shark 30): Similar to behaviour #1, but this shark was generally absent during September-November.
3. Highly absent across the year (*i.e.,* Shark 31): This shark was detected for a period longer than 2 years. It showed 50% occurrence probability at the beginning of the year and reduced in September, being rarely detected the rest of the monitoring period, especially in June (30–0%).

From the individual-level plots (shown in Fig. 7) there are a few binned residuals (see in Fig. S1) that are more extreme than would be expected by the model as there are certain patterns. In particular, three of the four sharks with monitoring periods >1 year, they did not exhibit the same occurrence patterns year after year, and this is not accounted for by the model. While there are many ways in which to extend the modelling structure to account for a variety of different patterns, we leave these analyses out for future work when more data across sharks over a longer period is available.

**Figure 8:**
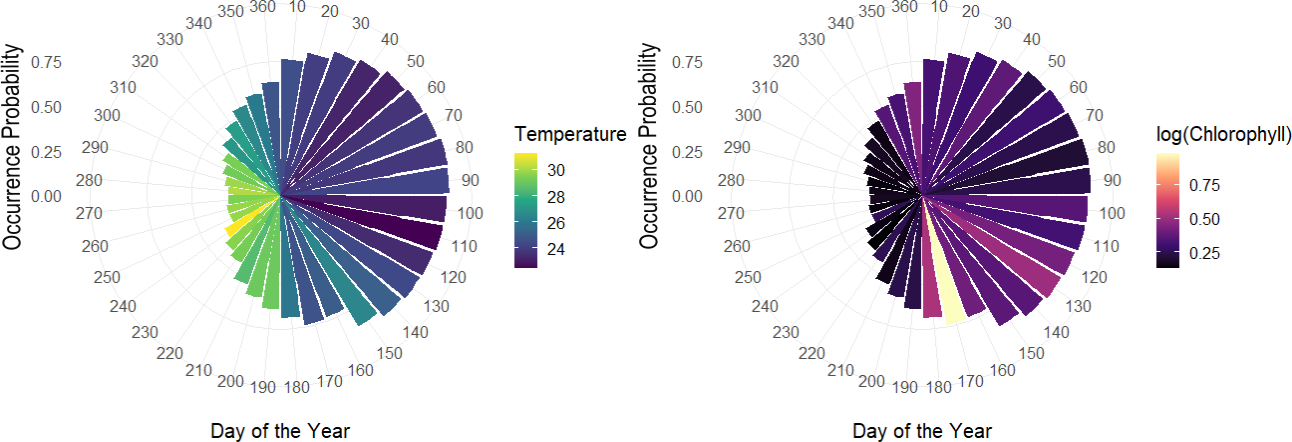
Polar coordinate plot using the pointwise estimates of the population-level occurrence probabilities over the course of the year. Larger bars reflect higher periods of occurrence probabilities and colours denote average values of temperature throughout the given month of the year (left). Same as left with colours relating average values of chlorophyll for a given month of the year during the study period (right).

The overall effect of each variable in the hierarchical model, showed that day of the year had an important effect, especially based on the sine term, where the highest occurrence probabilities are expected from April–June and the lowest from September–October. According to the logarithm scale of chlorophyll, the effect was negative for almost all the individuals indicating that concentration of Chla approximately less than logarithm 2.7 on the original scale led to higher occurrence probabilities. Meanwhile, although temperature tends to be correlated with the seasonality terms in the model related to day of the year, some of the individual-level coefficients associated with temperature varied, either positively or negatively (Fig. 8).

Among the expected values for the population level effects, *μ*, the seasonal terms in the model seem to be a driver of occurrence patterns in bull sharks, in particular the sine term in the model [95% CI for *μ*_2_:(1.74, 2.82)], which corresponds to higher occurrence periods in the spring and lower occurrence probabilities in the fall, as well as the effect of log (chlorophyll) [95% CI for *μ*_4_: (−0.64, −0.01)]. While the CI for the effect of temperature contains zero, [95% CI for *μ*_3_:(−0.11, 0.17)], the values of temperature centred at 28 °C were correlated with the sine term in the model (*r* =  − 0.81), as temperature naturally fluctuates steadily across the course of the year. As such, variations in temperature, after accounting for what is already explained by the terms for day of the year, it is not expected to be a driver of occurrence. Moreover, we can see that the population-level occurrence probability over the course of the year does in fact vary across values of temperature, with lower temperatures generally related to higher occurrence probabilities and higher temperatures to lower occurrence probabilities (Fig. S2).

To assess the model, we computed pooled ‘binned’ residuals, as discussed in Gelman et al. (2013) using 100 bins. Using replicate draws *y*_*rep*_ from the posterior predictive distribution, we also constructed 95% credible intervals in order to compare whether the ‘average residuals’ were extreme relative to what the model assumes they should be. As seen in Fig. S1, most of the residual values fall within the 95% CI, although there are a few that fall outside of this range. The fitted model appears to capture much of the variation in the data except for a few events.

## Discussion

Using a hierarchical logistic regression model, with inference conducted in a Bayesian framework, had clear advantages to investigate the habitat use of the bull sharks in CPNP. It allowed us to determine (1) the differences by sex and length, and (2) how certain environmental factors driven certain behaviors (Carpenter et al., 2017). One of the other advantages of using a hierarchical model is that it was possible to estimate the occurrence probability curves to each shark.

It is important to consider that tracking bull sharks in CPNP had some limitations. The predictions from acoustic data underestimate the utilization of areas with few (or no) receivers and overestimate areas with many receivers (Whitney, Papastamatiou & Gleiss, 2012). The range tests were conducted in a single type of sea condition: calm and steady. Stormy days in the fall could affect the detection coverage, increasing the noise, then more testing it is recommended. Although we weighted probabilities, the probability of an animal being detected is not going to be linearly related to the number of receivers. We did have some acoustic coverage of the pelagic environment (deep receivers), but our analysis was mostly restricted to coastal environments.

Despite the limitations, the study design was capable of register differences according to the total length of bull sharks. Those could be related to ontogenetic changes in what they feed upon (Matich, Heithaus & Layman, 2011). Studies shows that juveniles of various shark species commonly exhibit strong site fidelity, and that larger sharks tend to move over much wider areas to find larger prey (Karl et al., 2011; Brunnschweiler, Abrantes & Barnett, 2014; Bangley et al., 2018). Werry et al. (2012) found that with increasing body size, movements by bull sharks between different habitats also increase. The 32 bull sharks varied by sex and length, where juvenile females had the highest probably of being present, whereas adult males the lowest.

Using the hierarchical model, we were able to determine the individual occurrence patterns, and post-hoc identify four groups: The first one, with reduced occurrence during September-November was the most common one and we believe that it was related to the seasonality and environmental variables. The second group showed a high occurrence during the first months of the year followed by a general period of absence, this pattern and the fourth group (no clear pattern due to the short monitoring period) could be related to the fact that the sharks were tagged externally, and the tags probably fell off or were removed by other sharks. In the third behaviour group, sharks are highly resident the whole year, but even they are present during September-November, their occurrence probability is lower.

The behavioral variability among the sharks described in this study may be product of several factors, including genetic variations, individual experiences, learning processes, and environmental influences (Clua & Linnell, 2019). For example, the genetic variations may contribute to differences in behavior by influencing an individual’s predisposition to certain traits (Vaudo et al., 2014). However, environmental factors also play a significant role in shaping behavioral variability. Experiences during early development, social interactions, cultural influences, and learning opportunities can all contribute to the diversity of behaviors observed within a population (Clua & Linnell, 2019).

We observed individual variability, responding differently to the seasonal probability to be detected, both sexes are likely to be present in April, whereas by October both sexes are not as likely to be present, although the year-round occurrence probability for females is larger than males. Previous studies suggests that these particular differences can be related to the reproductive response, some females moving to another habitat to give birth, but the rest of the population do not, this phenomenon is called “partial migration” (Papastamatiou et al., 2013; Mendiola, 2015).

The behavioral variability is often seen as adaptive because it allows individuals to respond flexibly to changing environmental conditions. It promotes the exploration of new strategies and behaviors, which can increase the chances of survival and reproductive success in diverse and unpredictable environments (Vaudo et al., 2014). When we compare the probabilities across size and sex in October, and other times of the year, large females are 30% more likely to be present than the males. In general, differences across sex are much higher from July to beginning of January, whereas in the spring, both sexes behave much more similarly. Females from other species such as the scalloped hammerhead shark *Sphyrna lewini* segregate from males by moving to an offshore habitat to feed on different, more energy-rich prey which enables increased growth rates, and this is necessary to support the embryos development (Klimley, 1987; Hoyos-Padilla et al., 2014).

The sexual segregation for some shark species may be a strategy to increase female fitness by reduced mating (Wearmouth & Sims, 2008; Papastamatiou et al., 2013; Espinoza et al., 2016; Lara-Lizardi et al., 2022). In previous studies in Australia (Simpfendorfer & Tobin, 2015; Yeiser, Heupel & Simpfendorfer, 2008; Werry et al., 2012), juvenile bull sharks were known for displaying consistent use of estuarine habitats, despite variable environmental conditions. Simpfendorfer & Tobin (2015) determined that these areas were often highly productive habitats, and bull sharks may utilize these areas to increase survivorship through reduced predation and competition. CPNP is coastal and it is away from a freshwater input. This makes the Cabo Pulmo bull shark population quite different than those existing in estuaries.

The occurrence index showed that sharks were detected more than a third of the monitoring period, and were generally absent in October in all areas, which coincides with the highest temperatures. Previous studies have shown that *C. leucas* has a clear preference for coastal habitats and a seasonal occurrence (Snelson, Mulligan & Williams, 1984; Heupel & Simpfendorfer, 2007; Brunnschweiler, Abrantes & Barnett, 2014; Simpfendorfer & Tobin, 2015). Their presence at these sites could be also related to the high abundance of teleost prey concentrated there. For example, E. M. Hoyos-Padilla (pers. comm., 2019) found that bull sharks in the Mexican Caribbean (Playa del Carmen) arrive at reefs at the same time as the amber jacks (*Seriola dumerili*).

The day of the year has an important effect on occurrence probabilities, according to the hierarchical model. Although temperature tends to be correlated with the seasonality, some of the individual-level coefficients associated with temperature varied, either positively or negatively. Simpfendorfer & Heupel (2012) also demonstrated that temperature was significant in explaining the occurrence of young bull sharks, but only as a factor that may control seasonal cycles. Many aspects of bull shark physiology are regulated by temperature, which might explain the fine scale movement patterns and the close association with specific temperature ranges (Hearn, Ketchum & Klimley, 2009; Simpfendorfer & Heupel, 2012). The movements to maintain body temperature within an optimal range can be explained using the hypothesis of behavioural thermoregulation which states that fish occupy a thermal niche that maximises vital rates, such as growth, survival, and reproduction (Curtis, Adams & Burgess, 2011; Hammerschlag et al., 2012; Brunnschweiler, Abrantes & Barnett, 2014).

On the other hand, the effect of the chlorophyll was negative for almost all the individuals indicating that lower concentration of chlorophyll led to higher occurrence probabilities. As a top predator it is hard to argument that the primary productivity could determine the occurrence patterns directly. However, some fish species like the yellowtail amberjack, *Seriola lalandi*, that are bull shark prey, tend to aggregate and spawn when the chlorophyll is high (Sala et al., 2003; Lubitz et al., 2023).

Finally, bull sharks tend to be absent from the park two thirds of the year, leaving the protected area and making them vulnerable to non-selective fishing practices, such as trawling and purse seiners, which are often registered in the Gulf of California. Therefore, effective conservation and management efforts for bull sharks will require both local (within the Park) and regional (beyond the park) attention and cooperation (Karl et al., 2011).

## Conclusions

This study presents a comprehensive analysis of the seasonal occurrence and individual variability of bull sharks in Cabo Pulmo. By unravelling their migration patterns, habitat preferences, and individual behaviors, we contribute to the growing body of knowledge surrounding these predators, ultimately supporting their conservation and ensuring the long-term health of the marine ecosystems they inhabit.

Future research should focus on pregnant females close to parturition, neonates and juveniles, and their associated habitats and subsequent ontogenetic changes in habitat-use, which is still unknown in Cabo Pulmo. This would provide a more cost-effective and efficacious approach to long-term conservation given that the habitats utilized by neonate and juveniles are relatively restricted compared to adult bull sharks that occupy deeper and offshore waters within and beyond the boundaries of the marine reserve. Hence, neonates and juvenile bull sharks that occupy shallow and coastal waters are particularly vulnerable to the recent construction projects of marinas, hotels and houses near Cabo Pulmo National Park.

## Supplemental Information

10.7717/peerj.17192/supp-1 Supplemental Information 1 Supplementary Figures

10.7717/peerj.17192/supp-2 Supplemental Information 2 Raw dataset of bull sharks acoustically monitored Daily detections, temperature and chlorophyll.

## Additional Information and Declarations

### Field Study Permissions

The following information was supplied relating to field study approvals (i.e., approving body and any reference numbers): Field experiments were approved and was carried out under permits from the Secretaría de Agricultura, Ganadería, Desarrollo Rural, Pesca y Alimentación (DGOPA.06668.150612.1691) and Comisión Nacional de Áreas Naturales Protegidas, CONANP (F00.DRPBCPN-APFFCSL.REBIARRE-102/13) of Mexico.

### Data Availability

The following information was supplied regarding data availability: The raw measurements are available in the Supplementary Files.
