# Supplementary material for: Seasonal occurrence and individual variability of bull sharks, Carcharhinus leucas, in a marine reserve of the southwestern Gulf of California
Source: PeerJ. 2024 May 16;12:e17192. doi: 10.7717/peerj.17192 (PMC11102736; doi:10.7717/peerj.17192)
Supplement: Supplemental Information 1 [file peerj-12-17192-s001.docx]

**Supplementary material**

Supplemental Table 1. Sharks tagged in CPNP since 2015. Where Tag ID is the acoustic code, TL (total length in centimetres) and RI is the residency index.

| **ID** | **Tagging date** | **Tag ID** | **Sex** | **TL (cm)** | **RI** |
| --- | --- | --- | --- | --- | --- |
| CL1 | 20/11/2015 | 28990 | Unknown | 170 | 0.04 |
| CL2 | 11/05/2016 | 28983 | Female | 200 | 0.17 |
| CL3 | 12/05/2016 | 28986 | Male | 180 | 0.06 |
| CL4 | 12/05/2016 | 28987 | Female | 200 | 0.16 |
| CL5 | 12/05/2016 | 28991 | Male | 180 | 0.05 |
| CL6 | 12/05/2016 | 28996 | Male | 180 | 0.08 |
| CL7 | 29/05/2016 | 29000 | Female | 170 | 0.07 |
| CL8 | 02/06/2016 | 28993 | Male | 170 | 0.06 |
| CL9 | 02/06/2016 | 28997 | Female | 170 | 0.07 |
| CL10 | 28/05/2017 | 16317 | Male | 210 | 0.13 |
| CL11 | 05/05/2018 | 16845 | Male | 200 | 0.1 |
| CL12 | 05/05/2018 | 16846 | Female | 170 | 0.31 |
| CL13 | 05/05/2018 | 16850 | Male | 160 | 0.12 |
| CL14 | 05/05/2018 | 18707 | Female | 200 | 0.14 |
| CL15 | 05/05/2018 | 18708 | Male | 180 | 0.1 |
| CL16 | 06/05/2018 | 13044 | Female | 160 | 0.61 |
| CL17 | 06/05/2018 | 13046 | Male | 180 | 0.3 |
| CL18 | 06/05/2018 | 13048 | Female | 170 | 0.21 |
| CL19 | 06/05/2018 | 14666 | Female | 160 | 0.24 |
| CL20 | 06/05/2018 | 13042 | Female | 180 | 0.54 |
| CL21 | 06/05/2018 | 14668 | Male | 230 | 0.22 |
| CL22 | 06/05/2018 | 16849 | Female | 160 | 0.14 |
| CL23 | 12/05/2018 | 13924 | Female | 250 | 0.09 |
| CL24 | 12/05/2018 | 15900 | Male | 200 | 0.2 |
| CL25 | 12/05/2018 | 15901 | Female | 280 | 0.19 |
| CL26 | 13/05/2018 | 13925 | Female | 200 | 0.1 |
| CL27 | 13/05/2018 | 13926 | Male | 200 | 0.19 |
| CL28 | 13/05/2018 | 15902 | Male | 200 | 0.68 |
| CL29 | 12/05/2018 | 15903 | Female | 280 | 0.19 |
| CL30 | 13/05/2018 | 13927 | Female | 200 | 0.1 |
| CL31 | 13/05/2018 | 13928 | Male | 200 | 0.19 |
| CL32 | 12/05/2018 | 13929 | Male | 200 | 0.19 |


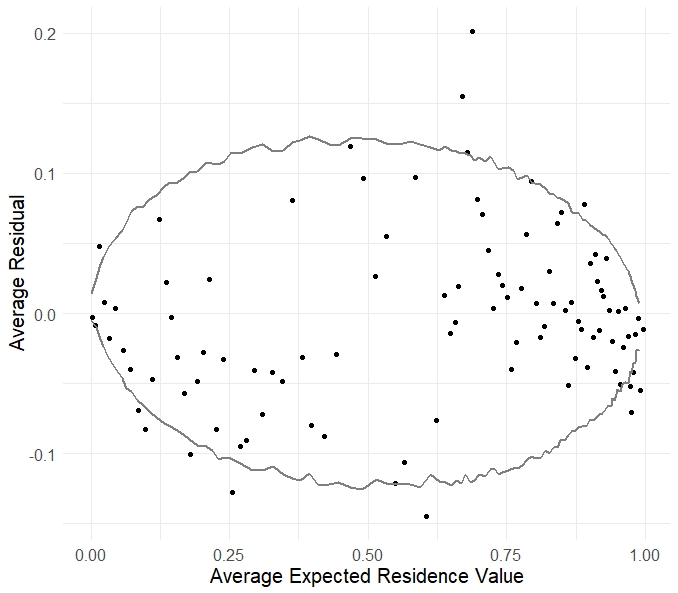


Supplemental Figure 1. Pooled binned residuals for the hierarchical logistic regression model along with 95% credible intervals (grey lines) constructed by using draws from the posterior predictive distribution.

**
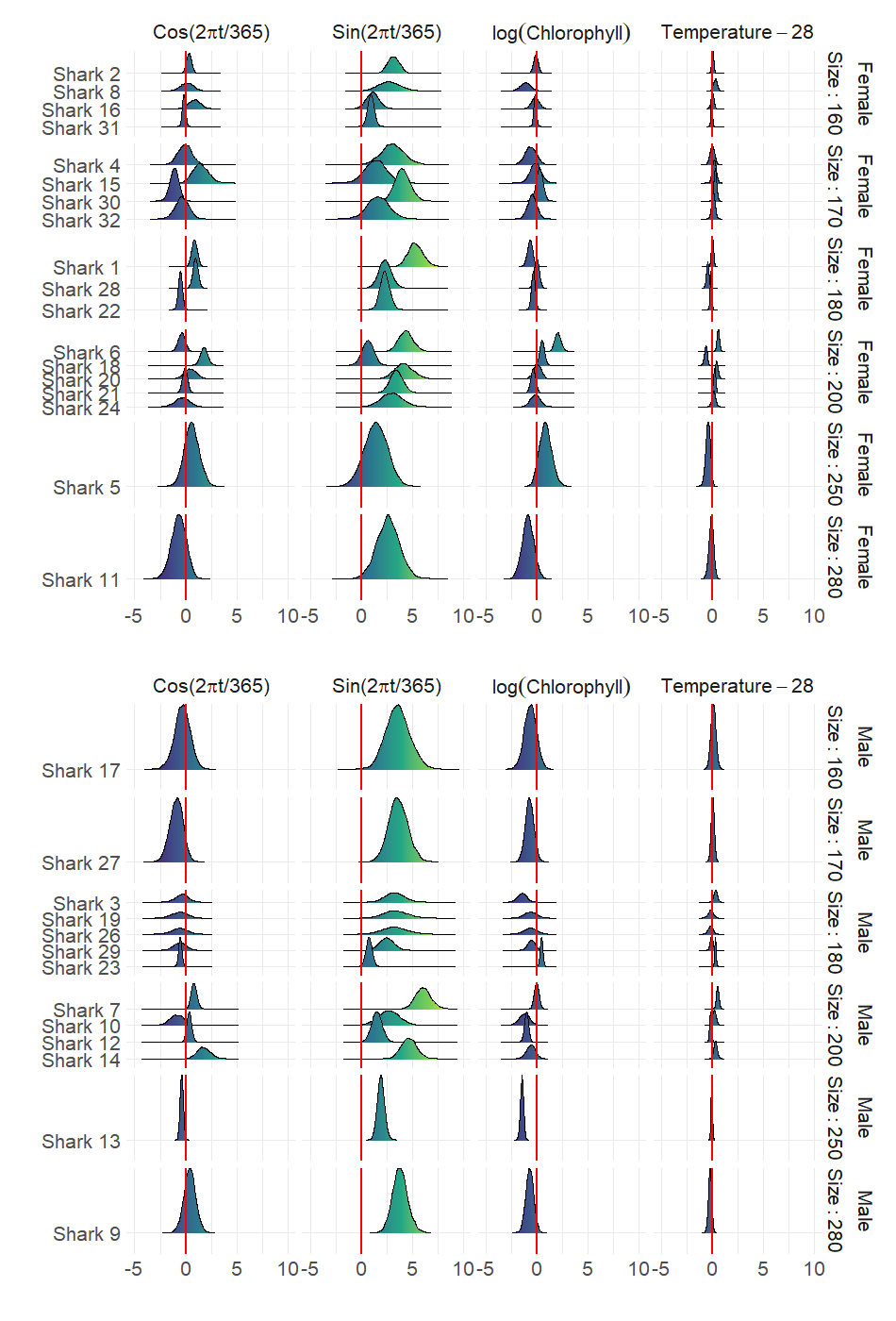
**

Supplemental Figure 2. Density curves of the marginal posterior distributions $p(\beta_{i,j}|\boldsymbol{y})$ for each individual. The red line at zero indicates the value of the effect that leads to an occurrence probability of 50%, were no other terms in the model included. Values to the right of the red line would lead to an occurrence probability greater than 50%, and vice versa for values to the left of the red line.
